# Extracorporeal Membrane Oxygenation in Severe Acute Eosinophilic Pneumonia

**DOI:** 10.3389/fmed.2019.00065

**Published:** 2019-04-10

**Authors:** Sean C. Dougherty, Sophia Ghaus, Orlando Debesa

**Affiliations:** Division of Pulmonary Disease and Critical Care Medicine, Department of Internal Medicine, Virginia Commonwealth University, Richmond, VA, United States

**Keywords:** acute eosinophilic pneumonia, ECMO, pulmonary eosinophilia, ARDS, bronchoalveolar lavage

## Abstract

Acute Eosinophilic Pneumonia (AEP) is a potentially fatal cause of hypoxemic respiratory failure characterized by fever, diffuse bilateral pulmonary infiltrates, and pulmonary eosinophilia. Shown to be associated with a number of environmental exposures and lifestyle choices, AEP has a good prognosis when diagnosed early and treated with corticosteroids. In this clinical case report, we detail the presentation, evaluation, diagnosis, and management of a 40-year old male who presented to the emergency department with dyspnea, chills, and diaphoresis. He had a history of pulmonary embolism 8 years prior but was otherwise healthy, though he had re-started smoking cigarettes a week prior to presentation. Initial chest CT scan revealed widespread mixed groundglass and solid airspace opacities; over the next 12 hours, he rapidly decompensated and after not responding to other invasive mechanical ventilation, was emergently cannulated for veno-venous extracorporeal membrane oxygenation (V-V ECMO). Bronchoalveolar lavage later revealed pulmonary eosinophilia, and after an infectious workup was negative, a diagnosis of AEP was reached and the patient was started on corticosteroids. To our knowledge, this is one of few published cases of AEP requiring V-V ECMO for clinical stabilization, highlighting the utility of this treatment modality in severe disease.

## Background

Acute Eosinophilic Pneumonia (AEP) is a rare, potentially fatal disease characterized by fever, hypoxemic respiratory failure, diffuse bilateral pulmonary infiltrates, and pulmonary eosinophilia. First described in 1989 (1), AEP generally affects young, otherwise healthy males (2, 3) and has been shown to be associated with cigarette use (either initiation or restarting of smoking) (2, 4, 5), environmental exposures (6, 7), and medications (8), however the exact etiology remains unknown. The worldwide incidence is also unknown, though a 2004 report describing AEP among US military personnel in Iraq estimated it to be 9.1 per 100,000 person years in this population (9).

Patients generally present with combinations of dyspnea, cough, and fever, though a myriad of other symptoms has also been noted (9). Since its initial description, multiple different diagnostic criteria for AEP have been proposed. The most widely accepted are those developed by Philit et al.; these include: (1) acute onset of febrile respiratory manifestations <1 month in duration, (2) bilateral diffuse infiltrates on chest radiograph, (3) hypoxemia defined as PaO_2_ <60 mm Hg or arterial oxygen saturation <90% on room-air pulse oximetry, (4) bronchoalveolar lavage (BAL) showing >25% eosinophils or eosinophilic pneumonia on lung biopsy, (5) absence of known causes of pulmonary eosinophilia, including drugs, toxins, and infections (10).

Approximately two-thirds of patients diagnosed with this disease require invasive mechanical ventilation (11). Though often mistaken for acute respiratory distress syndrome (ARDS) or severe community acquired pneumonia on initial presentation, AEP has a good prognosis when diagnosed early and treated with corticosteroids, as prompt clinical improvement is generally noted within 48 h of initiation of steroids (1, 11, 12).

Veno-Venous extracorporeal membrane oxygenation (V-V ECMO) is a potentially lifesaving treatment providing protective ventilation in cases of refractory hypoxemia. V-V ECMO is commonly used in the setting of ARDS and elsewhere within the intensive care unit as it limits the inherent risks of lung injury associated with invasive mechanical ventilation (13, 14). Recently, Combes et al. proposed three criteria for initiation of ECMO in patients that had undergone endotracheal intubation and been receiving ventilation for <7 days; they include: (1) a ratio of partial pressure of arterial oxygen (P_a_O_2_) to the fraction of inspired oxygen (FiO_2_) of <50 mm Hg for >3 h, (2) a P_a_O_2_:FiO_2_ of <80 mm Hg for >6 h, or (3) an arterial blood pH of <7.25 with a partial pressure of arterial carbon dioxide of ≥60 mmg Hg for >6 h, with the respiratory rate increased to 35 breaths per minute and mechanical ventilator settings adjusted to keep a plateau pressure of ≤32 cm of H_2_O and despite ventilator optimization (15). V-V ECMO has also been used in the setting of AEP; there are currently five reported cases of severe disease requiring V-V ECMO with zero reported fatalities, thus displaying the utility of this treatment modality in patients with AEP, though a survivorship bias may be present in the literature (12, 16–19).

In this clinical case report, we detail the presentation, evaluation, and diagnosis of a severe case of AEP, highlighting the effectiveness of V-V ECMO following rapidly decompensating hypoxemic respiratory failure and shock, in turn expanding upon the current literature describing this method of treatment in AEP.

## Case Presentation

A 40 year-old male presented to an outside hospital with 3 days of persistent dyspnea, non-productive cough, chills and diaphoresis. He denied fever but also noted intermittent, non-radiating, burning chest pain and an unpleasant taste in his mouth. His past medical history was significant for pulmonary embolism upon returning from military deployment in Afghanistan 8 years prior. He was no longer on Warfarin, however had been taking Ibuprofen, 1,600 mg by mouth twice daily for the last 48 h. He was a former smoker who had recently re-started smoking again within the last week after over a year of abstinence. He was previously incarcerated though was released from prison ~1 year prior to admission and now worked as a construction worker. He had not recently traveled and had no pets or sick contacts at home.

**Figure 1:**
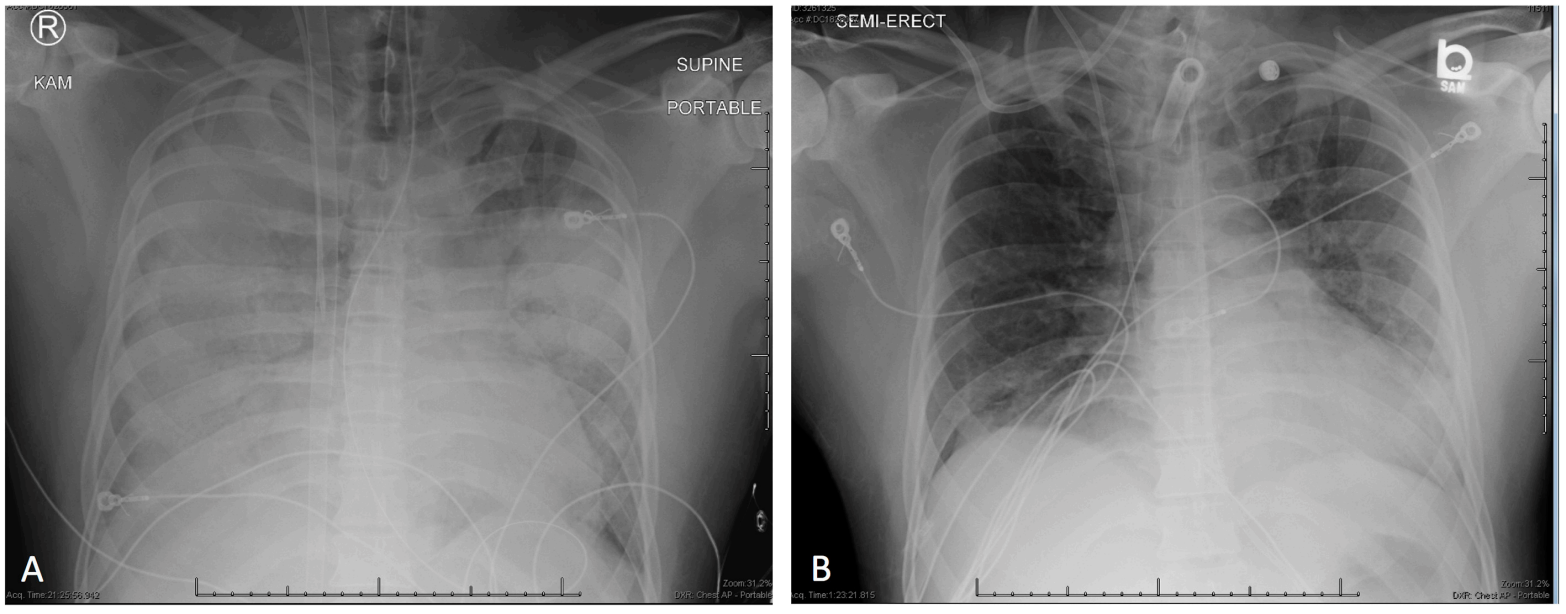
**(A)** Anterior-Posterior chest radiograph on hospital day 1 showing diffuse consolidative airspace disease. Radiograph was taken immediately following ECMO cannulation after transfer to our tertiary care facility. **(B)** Anterior-Posterior chest radiograph on hospital day 12 (4 days prior to discharge) showing decreased opacification of both the middle and lower lung zones with marked improvement of degree of airspace disease.

**Figure 2:**
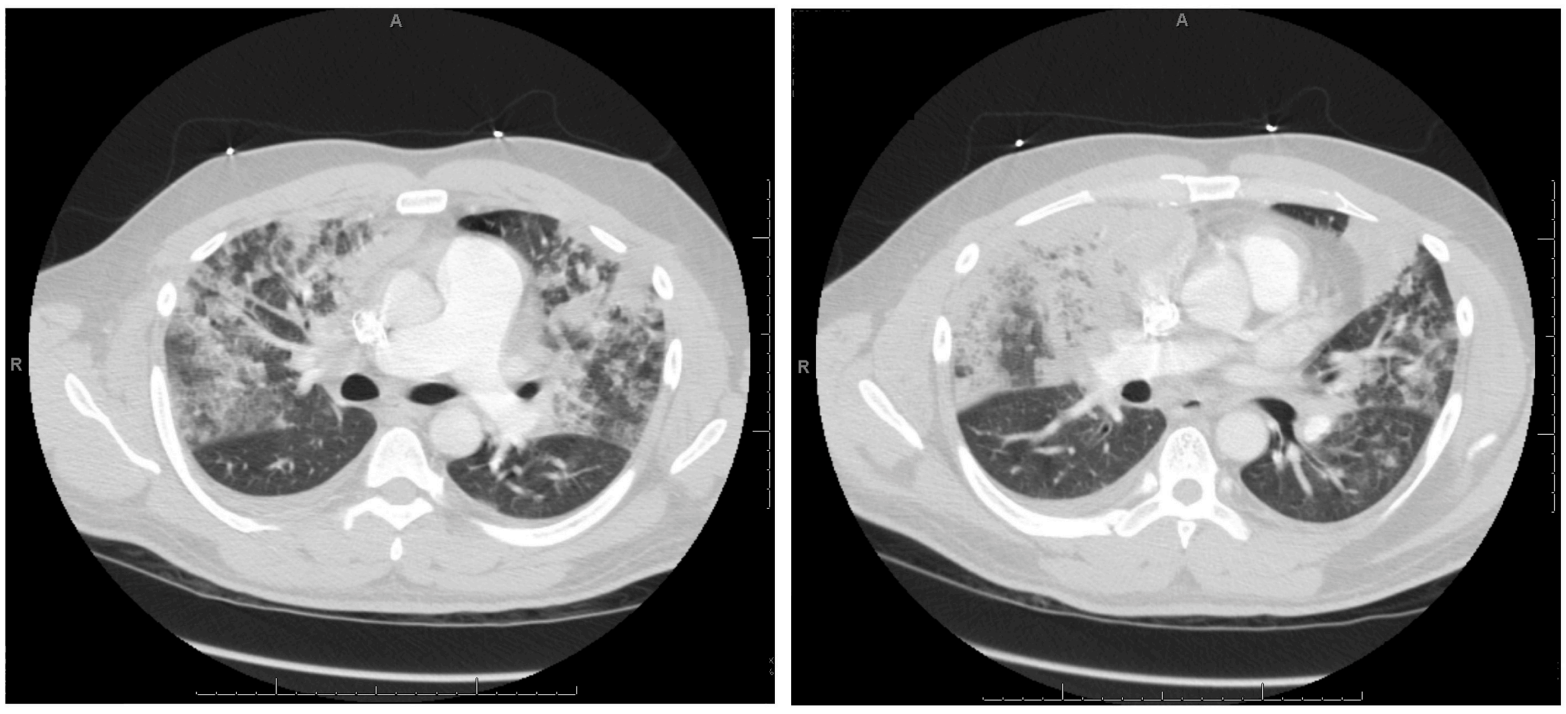
Axial Chest CT angiography from hospital day 0 showing bilateral, extensive mixed groundglass and solid airspace opacities predominantly in the middle lung zones without evidence of pulmonary emboli.

On admission, vital signs were significant for a temperature of 38.2°C, heart rate of 123 beats per minute, blood pressure of 123/88 mm of Hg, respiratory rate of 32 breaths per minute, and peripheral capillary oxygen saturation of 94%. He was 186 centimeters tall and weighed 110 kilograms. Physical examination was notable for diminished breath sounds bilaterally, though the patient was not in respiratory distress or wheezing. There was no lymphadenopathy, no calf tenderness and no rash present. The patient was alert and oriented, though anxious appearing. Laboratory values were significant for a white blood cell count of 15.4 × 10^∧^9/L with 84% segmented neutrophils, 8% lymphocytes, 7% monocytes and 1% eosinophils. Complete metabolic panel and D-dimer were within normal limits. Chest radiography revealed bilateral pulmonary infiltrates (Figure 1A) and chest CT revealed widespread mixed groundglass and solid airspace opacities predominantly in the middle lung zones without evidence of pulmonary emboli (Figure 2). Piperacillin/Tazobactam, Vancomycin, Azithryomycin, Micafungin, and Bactrim were empirically initiated.

Over the course of the next 12 h, the patient began experiencing refractory hypoxemia. He was placed on a non-re-breather mask, then high-flow nasal cannula and then emergently intubated for ongoing hypoxemia. Arterial blood gas following intubation was significant for a pH of 7.20, PCO_2_ of 54, PO_2_ of 60 and bicarbonate of 20.8. Ventilator settings at that time included a positive end expiratory pressure of 22 cm H_2_O, fraction of inhaled oxygen at 100%, peak inspiratory pressure of 44 cm H_2_O, tidal volume of 860 mL, and respiratory rate of 28 breaths per minute. The PaO_2_/FiO2 was 65. In the setting of profound hypotension and acute hypoxic respiratory failure, the patient was then transferred to our tertiary care facility for emergent V-V ECMO; cannulation of the right internal jugular vein with a 31 French dual lumen cannula was performed at the bedside. Immediate improvement in pulse oximetry was noted at an ECMO blood flow of 4.6 liters per minute, fraction of membrane lung oxygen at 100%, sweep gas flow of 5 L/minute and 3,800 rotations per minute. The patient was started on stress dose Hydrocortisone, 50 mg IV every 6 h, in the setting of distributive shock secondary to presumed adrenal insufficiency, Norepinepherine at 0.3 micrograms per kilogram per minute, and Vasopressin at 2.4 units per hour. White blood cell count upon transfer was measured to be 38.4 × 10^∧^9/L, though a cell differential was not drawn at that time.

**Figure 3:**
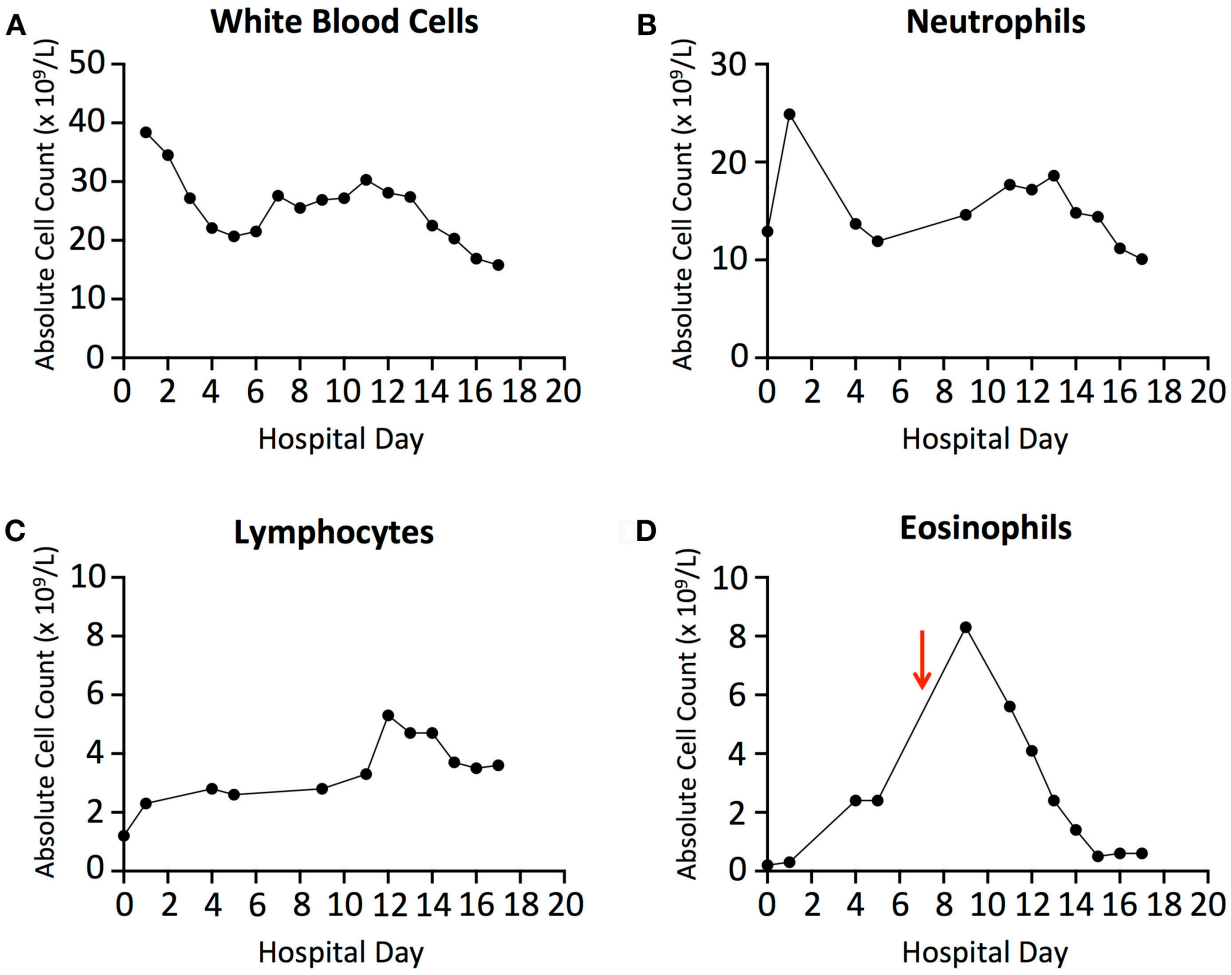
**(A)** Absolute White Blood Cell count trends throughout hospitalization. **(B)** Absolute Neutrophil Count trends throughout hospitalization. **(C)** Absolute Lymphocyte Count trends throughout hospitalization. **(D)** Absolute Eosinophil Count trends throughout hospitalization. Red arrow denotes initiation of Prednisone on Day 7.

Bronchoalveolar lavage (BAL) was performed 1 day later and notable for copious amounts of thin, green liquid without evidence of pus, bilaterally. Cellular analysis revealed a total nucleated cell count of 380 cells/mL with marked eosinophilia at 21% eosinophils (absolute cell count, 80 cells/mL), 4% neutrophils (15 cells/mL), 38% lymphocytes (144 cells/mL), 1% basophils (4 cells/mL), 6% macrophages (23 cells/mL), and 30% alveolar pneumocytes and bronchial cells (114 cells/mL). Complete blood count then revealed ongoing leukocytosis (18.6 × 10^∧^9/L) with evidence of peripheral eosinophilia (12.8%, 238 cells/mL) (see Figure 3 for complete blood cell count trends). IgE levels were also found to be elevated at 1,434 IU/mL. Extensive infectious and autoimmune work-ups were performed and found to be unrevealing; an HIV test was also negative.

While the work-up of this patient's acute hypoxic respiratory failure was ongoing, stress-dose hydrocortisone was continued. His clinical condition began to improve over the next several days; he was weaned from V-V ECMO prior to being weaned from invasive mechanical ventilation, with the fraction of membrane lung oxygen, blood flow, and sweep gas flow on ECMO all decreased gradually. A diagnosis of AEP was reached after the infectious work-up was confirmed to be negative and all other causes of pulmonary eosinophilia were ruled out. Piperacillin/Tazobactam was continued for 10 days empirically; all other antimicrobials were discontinued on day 6 of hospitalization. He was decannulated from V-V ECMO and started on Prednisone, 60 mg by mouth daily on day 7 of hospitalization. Peripheral eosinophil counts continued to downtrend while on corticosteroids (Figure 3D). He underwent tracheostomy on day 4 of hospitalization, was weaned off of vasopressors and mechanical ventilation on days 3 and 8 (respectively), transferred out of the intensive care unit on day 12 (Figure 1B), and eventually discharged home on day 16. He was continued on Prednisone, 40 mg by mouth daily until follow-up with Pulmonology 2 weeks after discharge.

## Discussion

Acute Eosinophilic Pneumonia is a potentially life-threatening cause of acute hypoxic respiratory failure. It has been shown to be associated with a number of drugs, alterations in smoking habits and environmental allergens, however the exact etiology remains unknown. Here, we have detailed the presentation, evaluation, diagnosis and management of a severe case of disease requiring V-V ECMO and vasopressors for clinical stabilization in the setting of distributive shock.

The obtainment of a thorough medical and social history revealed that this patient had a number of exposures previously shown to be associated with AEP. He had recently re-started smoking cigarettes within 1 week prior to admission after over a year of abstinence, worked as a construction worker on a crew tasked with performing roadwork, and had been ingesting a significant amount of Ibuprofen (1,600 mg by mouth twice daily) for 48 h prior to hospitalization. Current data showing associations between exposures and AEP is often limited to single case reports or small case series; however, alterations in smoking habits have been the most supported through epidemiologic and experimental data (2, 9). Cigarette smoke challenge tests have further been used to confirm the role of this inhalational exposure in AEP (20).

Our patient presented with multiple clinical features similar to those previously described (10). Namely, he was a young, otherwise healthy male, had acute onset dyspnea for 3 days prior to presentation, fever, diffuse bilateral pulmonary infiltrates on chest radiograph and groundglass opacities on CT scan (21), and absence of other known causes of pulmonary eosinophilia, such as infection. Despite having only 21% eosinophils on BAL, technically below the diagnostic threshold of 25%, we believe that the Hydrocortisone administered on admission and for 24 h prior to bronchoscopy resulted in lower levels of eosinophils. Hydrocortisone has previously been shown to negatively affect eosinophil chemotaxis, adherence, and result in eosinopenia (22, 23), which could explain the observed eosinophil percentages on BAL in our patient. In the absence of other more likely diagnoses and in the setting of the clinical presentation and severity of disease, we stand by a diagnosis of AEP.

The pathophysiologic mechanism by which cigarette smoke inhalation results in AEP has not been completely elucidated. Cigarette smoke is known to result in bronchial damage through a number of causes, including direct toxicity to bronchial epithelium (24, 25), oxidative damage (26), and recruitment of pro-inflammatory cells (27, 28). Increased production of proinflammatory cytokines and chemokines, including interleukin (IL)-6, IL-8, and tumor necrosis factor alpha, with resultant recruitment of activated macrophages and neutrophils to the lungs, is also associated with cigarette smoking (29–32). Previous studies have shown an increase in IL-8 levels and circulating neutrophils in peripheral blood in the early stages of AEP, followed by an increase in circulating eosinophils at a later time (6, 33, 34). De Giacomi et al. recently reported that peripheral eosinophilia was even more likely to be absent on admission in patients with smoking-related AEP (35); this trend was also observed in our patient.

The recruitment and survival of eosinophils in AEP is also thought to result from release of multiple cytokines and chemokines, including IL-5, IL-33 and others. IL-5 levels have been shown to be elevated in the vast majority of cases of AEP and result in increased eosinophil proliferation and release of proinflammatory mediators (36, 37). IL-33, a potent activator of eosinophils and basophils, has been reported to be significantly increased when measured in both serum and BAL and compared to patients with ARDS (38–40).

Corticosteroids are the mainstay of treatment in AEP, though there is currently no consensus on dosage or treatment duration. Previous reports have administered intravenous Methylprednisolone ranging from 60 to 1,000 mg daily in divided doses for severe cases of disease requiring invasive mechanical ventilation, with transition to oral Prednisone, 40–60 mg daily when the patient is extubated and tolerating medications by mouth. A steroid taper duration ranging from 3 days to 3 months was reported in these patients (1, 6, 9, 10, 41, 42). A recent retrospective study by Rhee et al. found the efficacy of a 2-week course of corticosteroids to be similar to that of a 4-week course, though the tapers were not adjusted for disease severity (43). Jhun et al. also recently highlighted the utility of initial peripheral eosinophilia in determination of corticosteroid taper, noting that patients who have evidence of initial eosinophilia generally have more mild disease and can undergo rapid taper (44). In our patient, given the severity of his clinical presentation and absence of peripheral eosinophilia initially, we elected for a 2-week taper. Of note, multiple patients have been observed to improve in the absence of corticosteroid administration (2, 6, 9, 10, 45), though these cases were reportedly less severe and often did not progress to respiratory failure.

Along with corticosteroid use in the treatment of AEP, this case demonstrates the utility of using V-V ECMO in patients with severe disease leading to respiratory failure refractory to mechanical ventilation. Our patient was placed on V-V ECMO upon arrival to the cardiac surgery intensive care unit due to refractory hypoxemia despite aggressive intervention with mechanical ventilation. This was an atypical application of V-V ECMO, as indications for ECMO are typically reversible conditions including ARDS due to severe bacterial, viral, or atypical pneumonia or aspiration, traumatic lung injury, awaiting lung transplant, and respiratory distress due to asthma (46). Benefits of using ECMO must be balanced with the consideration of potentially severe complications, including clotting within the cannulas, air bubbles within the drains, bleeding, premature decannulation, and risk of pneumothorax. Our patient was placed on a bivalirudin drip to maintain a PTT of 70, and did not experience these complications.

It should be noted that other less invasive and cheaper therapies need to be considered prior to initiation of V-V ECMO, especially in centers with limited access to ECMO. As shown by Papazian et al. in the ACURASYS trial, utilization of the neuromuscular blocker cisatracurium besylate is one such option, as it improved the adjusted 90-day survival rate, among other outcomes, in severe ARDS (47). Another minimally invasive option is prone positioning; Guérin et al. showed that patients with ARDS and severe hypoxemia who were placed in the prone position for at least 16 h straight had significantly decreased 90-day mortality compared to patients placed in the supine position (48). Despite our group not implementing these treatment modalities prior to initiation of ECMO, we recommend justification of V-V ECMO only once all other practical therapies have been exhausted.

The implementation of V-V ECMO early in this patient's hospital course allowed for the use of ultra-protective ventilation, with tidal volumes at 1.9 ml/kg (ideal body weight) while PEEP was maintained at 12 cm H_2_O. The combination of low tidal volumes with high PEEP levels has been suggested as an important strategy to reduce atelectrauma, which is defined as intra-tidal alveolar opening and closing, alveolar strain, and reabsorption atelectasis from oxygen lung toxicity (49). Low tidal volumes set to 6 mL/kg have been found to reduce alveolar epithelial and lung endothelial injury, reducing pulmonary edema in a rat model of acid-induced lung injury (50). A randomized clinical trial from 2000 found a 22% decrease in mortality associated with initial tidal volumes of ~6 ml/kg compared with 12 ml/kg (51) and the use of tidal volumes below 6 mL/kg has been associated with increased survival in patients with ARDS (52), indicating the potential for further study of the use of this ventilation strategy in patients with AEP. V-V ECMO prevents hypercapnea, which is typically one of the major concerns in these settings.

## Concluding Remarks

In summary, acute eosinophilic pneumonia is a rare, potentially life-threatening cause of acute respiratory compromise and hypoxemia that can be mistaken for ARDS or severe community acquired pneumonia. Though peripheral eosinophilia is often absent in the early stages of disease, a high index of clinical suspicion, thorough medical and social history and prompt BAL revealing >25% eosinophils should allow for diagnosis of disease. V-V ECMO should be considered in cases of AEP in which other types of invasive mechanical ventilation and rescue therapies are inadequate, as it allows for protective ventilation during administration of steroids until clinical stabilization is achieved.

## Ethics Statement

All clinical data in this case report was collected with the consent of the patient. A written informed consent was obtained from the patient for participation in the study and the publication of this report. The case report is exempt from institutional review board approval.

## Author Contributions

SD wrote the manuscript under the mentorship of OD and aided in the diagnosis of disease, SG helped write and edit the manuscript and aided in the diagnosis of disease, OD supervised case report formulation, acquired and interpreted clinical data, aided in the diagnosis of disease and was the physician of record.

### Conflict of Interest Statement

The authors declare that the research was conducted in the absence of any commercial or financial relationships that could be construed as a potential conflict of interest.

## Abbreviations

AEP: Acute Eosinophilic Pneumonia
ARDS: Acute Respiratory Distress Syndrome
BAL: Bronchoalveolar lavage
FiO_2_: Fraction of inspired oxygen
IL: Interleukin
PaO_2_: Partial pressure of arterial oxygen
V-V ECMO: Veno-Venous Extracorporeal Membrane Oxygenation.
